# “Unrigging the support wheels” - A qualitative study on patients’ experiences with and perspectives on low-intensity CBT

**DOI:** 10.1186/s12913-019-4495-1

**Published:** 2019-10-09

**Authors:** Elisa Haller, Nicole Besson, Birgit Watzke

**Affiliations:** 1 Department of Clinical Psychology and Psychotherapy Research, University of Zurich, Zurich, Switzerland; 2 Department of Psychology, University of Zurich, Binzmühlestrasse 14/16, Zurich, Switzerland

**Keywords:** Mental health services research, Client experiences, Acceptance, Telephone-CBT

## Abstract

**Background:**

Low-intensity treatments imply reduced therapist contact due to an emphasis on self-help and the use of technologies to deliver treatment. The role of the remoteness, the reduced therapist contact, and the interplay of these components has not been differentiated from a patients’ perspective so far. This study’s purpose is to capture patients’ experiences with telephone-based self-help cognitive behavioural therapy (tel-CBT).

**Methods:**

A subsample of mildly to moderately depressed patients (*N* = 13) who finished tel-CBT as part of a larger randomised controlled trial (RCT) in routine care were interviewed using a semi-structured questionnaire. Interviews were audiotaped, transcribed verbatim, and independently coded by two coders blind to treatment outcome. Using qualitative content analysis with deductive and inductive procedures, a two-level category system was established.

**Results:**

The category system contains four category clusters regarding expectations, self-help related aspects, telephone-related aspects, and implications for patients’ treatment pathway, and subsumes a total of 15 categories. Self-help related aspects circulate around the interplay between written materials and professional input, trust and support in the therapeutic relationship and its relation to the initial personal contact, as well as CBT principles. Telephone-related aspects entail perceived advantages and disadvantages of the telephone on an organisational and content level as well as a discourse around distance and closeness in the interaction. Although patients raised doubts regarding the long-term effect of the intervention on symptomatology, patients expressed satisfaction with the treatment and reported an immediate as well as a longer lasting personal impact of the treatment. These results indicate user acceptance with tel-CBT.

**Conclusions:**

This qualitative analysis captures patients’ experiences with tel-CBT and the perceived helpfulness of the diverse treatment components. This can facilitate refining aspects of low-intensity treatments and might improve dissemination.

**Trial registration:**

ClinicalTrials.gov NCT02667366. Registered on 3 December 2015.

**Electronic supplementary material:**

The online version of this article (10.1186/s12913-019-4495-1) contains supplementary material, which is available to authorized users.

## Background

The introduction of low-intensity treatments as one way to mitigate the overwhelming disproportion of demand and supply in evidence-based mental health services has been called a “revolution in mental health care” (Bennett-Levy et al., 2010, p. 3). The aim of this transformation is to improve access to evidence-based care by providing the least resource-intense care for common mental disorders. Low-intensity interventions entail a variety of different formats, modalities, and levels of intensity regarding duration and frequency of treatment as well as extent of therapist contact (Benett-Levy et al., 2010; [7]). Their commonality is the reduction of time and encounters between therapist and patient, by encouraging patients to manage their symptoms and by employing “health-technologies”. This is achieved through a) a shift to applying written and self-help materials, and b) the use of communication technologies (internet and telephone) intending to reach patients from a distance and to support therapists in conveying therapeutic content.

A growing body of literature supports the feasibility, acceptability, and efficacy of low-intensity treatments, including guided self-help cognitive behavioural therapy (CBT) [15, 18] and telephone-based interventions [4, 29]. Telemedicine has a long tradition in health care and the use of the telephone as a facilitator of guided self-help dates back to early studies on self-help interventions for depression (e.g., [10]). Increasingly, the telephone is recognised as a mediator of full psychological treatments with clinical effectiveness [4]. Telephone-delivered CBT (tel-CBT) as a stand-alone treatment normally comprises a highly structured treatment program based on core elements of CBT [23, 27, 33, 39]. Tel-CBT thus combines a low-threshold approach with the benefits of personal therapeutic support [34]. While the regular, scheduled sessions in tel-CBT might resemble traditional psychotherapy, the short sessions and the anchoring of the self-help approach in the treatment program imply reduced therapeutic input and augmented self-reliance on the patients’ side. Within the broad variety of low-intensity interventions, tel-CBT can be located on the higher end of the low-intensity spectrum due to regular therapist contact but a simultaneous strong emphasis on (guided) self-help.

Despite advantageous features and the promising results of low-intensity treatments, their uptake in routine care has been slow. With a few exceptions in the international context, where technology-mediated psychological treatments are delivered as part of a stepped care model (e.g., Improving Access to Psychological Therapies (IAPT) in the UK), traditional face-to-face therapies are prevailing the landscape of treatment provision models in many countries. One reason for a cautious implementation on a system level might be the relatively large dropout rates connected to low-intensity treatments. Attrition rates in some studies exceed those in face-to-face treatments [2]. This is particularly the case for internet-based psychological treatments with some unguided (internet-based) interventions having showed adherence rates of lower than 7% [12]. While dropout rates in self-help treatments vary widely [9] and are comparable to face-to-face treatments [15], there is contrasting evidence that attrition rates are lower in tel-CBT compared to face-to-face treatment (e.g., [28]), highlighting the capacity of the therapist-telephone combination to overcome barriers that could occur in face-to-face treatments. However, the therapists’ role is not only important for patient engagement and in ensuring larger adherence to treatment. The therapist-client relationship is considered a robust predictor of the therapeutic outcome. In psychotherapy research, a strong therapeutic alliance is deemed necessary and even responsible for therapeutic success (Horvath & Symonds, [20]; Norcross & Lambert, [30]). Telephone-delivered treatment is limited in that non-verbal cues such as gesture, facial expression, and eye-contact, which are considered important determinants of a therapeutic relationship, are missing (Haas et al., [19]). Clinicians fear that the lack of visual cues would curtail their ability to form an effective working alliance [32]. Yet, first empirical investigations show that the establishment of a working alliance in tel-CBT is equally possible relative to face-to-face CBT [35]. Although the therapist-client relationship might also be compromised by the reduced therapist contact in guided self-help treatments, there is evidence that the therapist-patient relationship is possible with minimal contact [15], suggesting that the relationship itself is essential rather than the intensity or frequency of the contact [22]. With respect to guided self-help interventions specifically, one meta-analysis demonstrates that professional guidance in self-help intervention leads to improved treatment outcome compared to pure self-help approaches [18]. This indicates that a certain level of therapist support is indeed necessary to achieve clinical benefit.

Qualitative research on patients perspectives on low-intensity interventions with patients with depression and anxiety disorders are thus far primarily concerned with the examinations of online and computer CBT [3, 17]. These studies have revealed both perceived advantages and disadvantages of the reduced therapist contact and highlight individual differences in assigning value on professional support. It is noticeable that only a segment of patients with certain characteristics, such as those patients taking responsibility for their treatment and inciting motivation for the treatment, appears to benefit from the low-intensity treatment format [6]. Macdonald et al. [24] examined expectancies of and experiences with a minimal intervention that served to bridge the time to starting regular psychotherapy. The patients’ narratives revealed that the minimal interventions’ focus on resolving symptoms was largely incompatible with the patients’ need for seeking insight into the cause of their current condition [24]. This result implies that the lower end of the intensity spectrum (in this case: up to four brief, 15-30 minutes long sessions in addition to the use of a written manual) might not suffice to fulfil expectancies of patients regarding treatment process and outcome. With regard to tel-CBT, there exists one extensive qualitative exploration of patients’ acceptance of remote treatment delivery [5], which concludes that a shared construct amongst patients of accessing professional help outweighs the conventional construct of personal encounter in mental health service provision [5]. This finding implies that patients easily adapt to the telephone as a treatment modality, largely driven by the potential of increased access to care. However, the role of the self-help approach, and the interplay between remoteness of care and the emphasis on patients` self-reliance remain unclear.

If low-intensity treatments are to be implemented broadly and in a sustained manner, it is important to identify unique aspects of this type of treatment format, including advantages and disadvantages of the type of treatment as well as the exploration of mechanisms of change of low-intensity CBT with a special focus on tel-CBT. Patients’ insights can thus help to refine components of a treatment. Given the large variety of treatment intensity in terms of extent of therapist support within the low-intensity interventions described above, qualitative results might help to differentiate the relative importance of the components involved by disentangling aspects of structured and guided self-help CBT from distinct properties of the telephone.

In view of the promising results regarding low-intensity interventions for patients with common mental health disorders, we aim at examining patients’ narratives on their experiences with a telephone-delivered guided self-help CBT. Our qualitative study was conducted alongside a randomised controlled trial (RCT) investigating tel-CBT compared to treatment as usual (TAU) in routine care. We conducted this process evaluation for two main reasons: 1) We are interested in patients’ evaluation of unique aspects of a low-intensity and tel-CBT in order to better understand both the role of the guided self-help approach, the telephone as a delivery mode, and their interplay; 2) Little is known about predictors and mediators of change in tel-CBT; an explorative methodology regarding subjectively helpful treatment elements and processes can help to generate hypotheses about mechanisms of change in process-outcome research. The overall aim is to understand patients’ experiences with the treatment that might inform treatment conceptualisation and dissemination.

## Methods

### Study context and Intervention

This study was designed as a qualitative process evaluation with N = 13 patients nested within a larger RCT investigating the effectiveness and cost-effectiveness of a tel-CBT compared to TAU plus a mini intervention involving regular text messages about depression. Further study information can be found in the study protocol of the trial [41]. The study was approved by the local Ethics Committee. Patients randomised to tel-CBT were invited to take part in the qualitative interview study in order to contribute their experiences with and their views on the treatment concept.

A total of 54 patients were recruited into the trial and randomised to either tel-CBT (n = 29) or TAU plus text messages (n = 25). Patient recruitment took place between January 2016 and August 2018 and followed a twofold strategy: Adult patients were either acquired by cooperating General Practitioners (GPs) of the Canton of Zurich or referred themselves to the study programme through newspaper reports and announcements. Trial inclusion criteria comprised a score between 6 and 15 on the Patient Health Questionnaire (PHQ-9) as well as a diagnosis of mild to moderate Depression according to the International Classification of Diseases (ICD-10). In addition, participants were required to provide written informed consent. Exclusion criteria were severe or chronic depression, suicidal ideation, participation in a psychological or psychiatric therapy at present or in the past 3 months, insufficient German proficiency, or physical or cognitive inability to complete questionnaires.

Eligible and consenting patients received a short-term CBT, the German adaptation of “Finding inner balance” ([33]; Tutty et al., [38]; [34]). The program comprises an initial face-to-face session and 8-12 subsequent, weekly and later biweekly scheduled telephone sessions of 30-40 minutes duration. The content is organised around four evidence-based elements of depression treatment (psychoeducation, behavioural activation, cognitive restructuring, and relapse prevention) and is provided in the form of a workbook for patients with 8 chapters. These include psychoeducational material, case vignettes, and exercise and homework sheets.

The therapy was delivered by one of four trained study therapists. Therapists were clinical psychologists, who were in advanced CBT training and had several years of experience in treating patients with depression. All therapists received additional training in the tel-CBT manual prior to the trial and monthly supervision from a senior researcher and clinician (BW) during the intervention period.

### Participants

The qualitative interview study took place between October 2017 and February 2018. Due to organisational reasons the time frame for the interviews was restricted to these months, implying that only patients of the RCT who had finished tel-CBT between October 2016 and February 2018 (i.e., whose termination of treatment took place in the previous 12 months before or within the interview period) could participate. Following a consecutive sampling strategy, all eligible patients (n = 14) were contacted and invited to participate in the interviews to describe their experiences with the intervention. One patient could not be reached, whereas all others were contactable and agreed to participate. Further 10 participants of the RCT who finished tel-CBT before October 2016 or after Februar 2018 could not be included in the interview study. One of these 10 patients was the only individual that dropped out from the RCT, because of a lack of time to undertake tel-CBT due to a new job situation (in-service training).

The interviewed sample is predominantly female (87%), highly educated, and the age ranges from 26 to 79. All participants had at least one previous depressive episode and all interviewed participants were self-referred to the RCT. Characteristics of the selected sample are displayed in Table 1. The sample does not differ from the overall RCT sample regarding clinical and socioeconomic characteristics. The only difference pertains the referral source: All of the interviewed patients were self-referred, whereas one third of the overall RCT sample was referred by a GP.

**Table 1:** Characteristics of the study sample

Participant	Gender	Age	Previous depressive episodes	Previous psychotherapy experience	Initial symptom severity
1	Female	≥66	≥3	yes	7-9
2	Female	≥66	1	yes	10-14
3	Male	46-55	1	yes	7-9
4	Female	46-55	1	yes	7-9
5	Female	46-55	1	no	7-9
6	Female	36-45	≥3	yes	10-14
7	Male	≥66	1	yes	7-9
8	Female	<35	2	no	10-14
9	Male	≥66	≥3	yes	≥15
10	Female	46-55	1	yes	7-9
11	Female	<35	2	no	≥15
12	Female	56-65	≥3	yes	≥15
13	Female	56-65	≥3	yes	10-14

*Note.* Initial symptom severity as measured with PHQ-9; 7-9 indicating mild, 10-14 moderate, ≥15 severe depression symptoms.

### Interview procedure

We conducted semi-structured interviews to assess patients’ experiences with and views on tel-CBT. The interview guide was developed by the study team and contained 16 open-end, inductive questions, which revolved around the themes *reason for starting tel-CBT*, *experience with telephone as a therapy medium*, *therapy structure and content, therapist and working alliance.* The interview guide started with broad questions and ended with the opportunity to express whatever patients wished. The interview guide was revised by a senior researcher (BW). The final version was tested in two training interviews and last changes in the exact wording of the questions were made thereafter. The English translation of the interview guide can be found in the supplementary materials (Additional file 1).

Interviews were conducted over the telephone by one member of the research team (NB), who was a graduate student in clinical psychology and was previously trained by senior researchers and clinicians of the study team in conducting the interviews. The interviewer was neither involved in the treatment that patients had received nor in the study procedure and had no knowledge of patients’ clinical history, treatment course and outcome, or personal information, apart from last name and contact details. All patients gave their written consent prior to the interviews. The interviews’ duration ranged from 30 to 60 minutes.

### Data analysis

The interviews were audiotaped and transcribed verbatim according to predetermined transcription rules by two research assistants. The interview data were subjected to a qualitative content analysis [26]. In accordance with current recommendations, data were analysed without prior knowledge of any trial outcomes in order to avoid any bias in interpretation [31]. The Software MAXQDA 2018 (Verbi Software, 2017) was used to assist the qualitative content analysis. A sequential model of inductive and deductive development of categories was applied whereby a category can be understood as a conceptual assignment to text-based codes. The coding units referred to statements of participants that convey a meaningful message. First, deductive categories based on previous empirical results and the themes of the interview guide were established in order to structure the content. Deductive categories mostly related to the category clusters. For example, one deductive category was labelled “Expectations towards treatment”. The next step was to perform an iterative process of establishing inductive categories by grouping and coding text passages using the first 6 interviews. Two researchers (EH, NB) independently established additional categories inductively and subsequently discussed their codings, which resulted in a first version of the category system. Emergent topics were elaborated on in later interviews, following an iterative process of data reading, data coding, and data collection. In order to increase agreement, category clusters and categories were modified until consensus was reached. Inter-coder reliability was then established with three subsequent calculations of the inter-coder agreement in assigning statements to the categories within the category system. After each calculation, categories were modified and re-structured until consensus was reached. The first calculation comprised 5% of all statements. No agreement was reached in 15% of those statements, mostly because of different codings made on subcategory level. Following this, the remaining interviews were coded and the resulting category system was again discussed and revised. For the subsequent second and third calculation of inter-coder agreement, 10% of all statements were coded and compared. In the last calculation, the two coders reached complete agreement in 68% of the codings, partial agreement in 98%, and no agreement in 6%. Partial agreement refers to a minimum of one congruent coding within the statement. Subsequently, a two-level category system with four category clusters and 15 categories was established.

## Results

The categories (c) were structured within four category clusters (C). These include: 1) expectations and fears towards the intervention, 2) aspects of guided self-help, 3) aspects of the telephone as a treatment delivery modality, and 4) conclusion and implications for treatment pathway.

In the following section the categories that emerged within the second, third, and fourth category cluster are explicated due to their significant informative content. They are illustrated with representative quotations, which have been translated from German into English. The first category cluster is described on the higher-ranking level, because the results on category level are of secondary importance for our research question.

### Expectations and fears towards low-intensity CBT

When patients were asked to think about their pre-existing expectations before the telephone-based therapy had started, most of them initially denied having had any specific expectations beforehand. Despite a general curiosity and simple interest in how the therapy was going to work out, they had rather neutral conceptions about the whole procedure. After inquiring further details, they reported expectations concerning therapeutic and content aspects of the therapy as well as the telephone as a therapy medium. Most patients were unfamiliar with the cognitive behavioural approach and were unsure about what the therapeutic content would include. Their understanding was that they will observe their thinking and behaviour and that one goal will be to adapt and change them. There was an underlying scepticism of whether this is achievable through a telephone-mediated therapy. The most commonly reported concern were the missing visual cues during telephone contact and as to whether the therapist would be able to acquire an adequate understanding of the patients’ feelings without any visual information.

*“Of course, in the beginning I have definitely thought … I mean, you cannot see each other … , it could be weird, it might not work or it might not help ( … ). That it might be less personal, because you cannot see each other, or that you don`t get into it as much.“* (P11)

### Aspects of guided self-help related to treatment process and outcome

The second category cluster (C2) contains patients’ accounts on specific aspects of the guided self-help CBT approach. Six categories were established and are outlined in the next section: the first four categories pertain the therapists’ role in the guided self-help CBT, while the last two revolve around CBT principles and structural aspects of guided self-help.

#### Initial personal therapist contact (C2c1)

The personal contact between patient and therapist served as a kick-off and was able to address initial concerns. Getting acquainted with the therapist was retrospectively considered important, although not necessary for all patients. The patients’ accounts revealed that the face-to-face meeting with the therapist encouraged trust between them and made the therapy feel more personal. For these patients, the trustworthiness proved beneficial in two ways: First, it enabled the patients to be more open and honest about their private matters. Second, it appeared as though the initial personal contact strengthened the patient’s therapy commitment.

*“I think it is better like this, compared to if we would not have seen each other at all. I don`t think that I would have engaged with the same openness and honesty with a complete stranger, or a completely unknown voice or with knowing only the voice.”* (P3)

*“Well, for me it was very important, because I always had a mental picture of that person, who was on the phone. And I think, if it would have been completely without the first personal contact, it would not have had the same meaning, actually. In any case I have a positive memory of the first interview and it was a good opening, definitely.”* (P7)

Patients also pointed out an initial appeal to the therapist, which was largely enabled through the initial, personal clinical interview. Getting to know the therapist made the therapy more personal and provided a basis for trust and security.

*“Somehow yes … it is not like: you call or you receive a call, but instead you somehow know … well I know where this person is calling from and who I am speaking with.”* (P4)

#### Trust and emotional support in the therapist-client relationship (C2c2)

Patients unanimously had a positive perception of their therapists, holding for all three therapists involved in the study. Highlighted qualities were sympathetic, considerate and empathetic. Patients generally embraced professionalism and warmth in the client-therapist relationship. Some patients considered the good therapeutic relationship and the open, non-judgmental discussions the most helpful aspects of their interaction with the therapist.

*“I simply felt comfortable and felt like I was being in safe hands in terms of having the feeling that I can open up, without it getting to anyone or that someone would make fun of it or … No, not at all, I felt comfortable and was able to open up. I always had the feeling that I could tell whatever I want and that she [the therapist] absorbs it, embraces it and reacts to it.”* (P11)

The non-judgemental atmosphere of the interactions with the therapist made the patients feel seen and heard, affording the patients’ impression that their problems are accepted, acknowledged and taken seriously. What appeared beneficial for these patients was the therapist’s recognition of their efforts and the fact that patients were sharing their success and insights with someone and felt vindicated.

*“[it is] important that for example one receives affirmation. When I say, I will do this or that. And that [the therapist] saw that from another perspective and could somehow validate me, or motivate me, and say: ‘Yes, of course it is good that you do that’.”* (P9)

In view of these narratives, it seems that the therapist successfully recognised and addressed patients’ needs despite physical distance. Although the therapist’s role within this tel-CBT was not considered equally essential by all patients, the therapist’s support was relevant for all patients to be able to immerse into the therapy contents.

#### Interplay between (professional) guidance and independent activity (C2c3)

Patients welcomed the close supervision and support from the therapist, particularly in the beginning phase of the treatment. In the course of treatment, patients increasingly recognised the workbook as a therapeutic tool with personal relevance. The workbook did evolve into some sort of a “personal guidebook” with long-lasting value.

*“ … that I could potentially have a look there in the future, like a recipe book: ´How was it, when I took my notes, and what did I do back then?’. I find that very helpful.”* (P9)

In particular, the textualisation of the spoken words during the therapy sessions helped to deepen the understanding of the contents and facilitated adequate preparations for the next session. Putting thoughts and experiences on paper enabled the patients to be more concrete about strategies and personal areas of concern and enabled a tangible treatment output.

*“I basically found it [the workbook] quite useful. Because if we would have only had a conversation, I can imagine that one would remain in a vague space, but if I still have the book afterwards … then I have something black on white; a support, or a possibility to engage in between sessions. I can then also elaborate the tool box more purposefully, when I have the book. I find it almost indispensable that there is a written support, or a written record of the topics.”* (P3)

All patients valued the pragmatic approach of being provided with the therapeutic content on one hand and the possibility of autonomously engaging with the elaborated content on the other hand. Notwithstanding the independence, patients were in overall need of the professional support through the therapist. It was the combination of independent activity and the input received by the therapist that appeared central. It appears as though the therapists’ contribution was needed for the contents described in the workbook to unfold.

*“With her together, yes, that was actually the solution to my problems. Or the approach … , in a way I could elaborate them by myself and I additionally received an input by a professional or further ideas and tips or affirmation as well. That was very helpful.“* (P9)

#### Therapeutic dialogue (C2c4)

The shared narratives about the therapeutic dialogue circulated around an appreciation that there was room for personal issues despite the strong structure of the therapy program. The set briefness and the structured agenda of the telephone sessions did not restrict flexible adjustments of the conversations to the patients’ needs.

*“I always had the feeling that she [the therapist] also makes time for the other things ( … ) I could always tell her what was difficult. This was extremely important for me, that there was also room for that. And I would say that sometimes the book – it was certainly always an adressed topic and I always did my homework, but it is not … well there was space for the other stuff as well.” (P12).*

Within the therapeutic dialogue, another therapist factor – the perceived competency of the therapist – was revealed, which was reflected in patients’ portrayals of helpful input, suggestions, and new perspectives provided by the therapist.

*“I think she also took stunningly many notes, because she often referred to previous statements of mine. ( … ) This was sometimes indeed illuminating. Which sometimes also contradicted itself, and then I obviously asked myself why. And I could then look at it together with her [the therapist] and I found that somehow … this is what I meant when I said she impressed me. When she said something I was not prepared for. And then I started thinking about it properly, focused on it and I found that always very thrilling, I have to say.”* (P8)

#### Cognitive behavioural principles (C2c5)

Patients valued the solution-focused and pragmatic approach of the intervention and noted that a few simple and memorable strategies suffice to attain improved mood. With regard to CBT-specific principles all patients provided specific examples of elaborated techniques and described a clear function of the acquired techniques. By paying deliberate attention to feelings and thoughts, it was possible to become aware of thoughts, emotions, behaviour, and the interplay, for example, between social activity and mood regulation:

*“...during this time, I completely shut myself away, I stopped socialising ( … ) well now I go out again but it really struck me that this withdrawal can be part of the depression and I am now paying attention to arranging appointments with other people.”* (P4)

Monitoring symptoms helped recognising that social withdrawal was part of the depressive symptomatology and encouraged patients to be mindful about fluctuations and triggers of (depressive) mood. While one patient perceived behavioural activation as the most helpful component (also due to its easy implementation), cognitive work formed the core of the treatment for almost all patients. This was reflected by shared accounts on concrete, individual cognitive techniques. One patient summarised this well by explaining to be *“able to think my way up to the top in a downward spiral.”* (P10)

#### Structure (C2c6)

The continuity of the sessions and the regular interaction with the therapist were considered crucial for progressing in the therapy and for improving the mental state. The structure provided stability and afforded a prospect of goal attainment.

*“It is like a suspension bridge. There are two ropes, one on each side, and the deck underneath and one can … one knows, this is the way, when it gets stormy and [ … ] I then just trusted in that I can walk this way … and that it helps me along.”* (P4)

The majority of the patients noted that the biweekly sessions allowed for more autonomous and independent engagement with the therapy content and with themselves. The rhythm provided opportunities to integrate therapeutic strategies into their daily lives by road-testing suggested strategies and by ascertaining the applicability of strategies in certain situation. While the intervals and the amount of sessions were accepted by most patients, they provided diverse suggestions for improving the strict structure and limited amount of contacts: One patient (P12), for example – despite being aware of it – was negatively surprised by the “sudden” change to biweekly periodicity; another patient wished for a more gradual and slow reduction of treatment sessions (P3), while a third patient would have appreciated the possibility of additional sessions (P7).

### Telephone-related aspects of therapy process and outcome

Three categories evolved inductively within the third category cluster (C3) regarding aspects related to the telephone.

#### Telephone as an advantageous delivery medium (C3c1)

Patients expressed advantages of the telephone-modality on a practical and executive level but also on a content level. With no need for co-location, the therapy becomes independent of time and location, which makes it more adaptable to different lifestyles and suitable for people with demanding jobs or family commitments. In addition, conducting therapy sessions at home in a familiar environment has the potential of increasing comfort in talking to the therapist. For most patients, the absence of visual cues contributed to that effect (see category “closeness vs. distance”). Perceived judgement and subconscious reactions to it in a personal interaction diminish, which increases focus on the essential.

*“I had the feeling that I was less distracted, almost more focused. When I am facing another person, for example, I always have a body language. Sometimes I get the feeling that I have to sit like this or in a certain way, or whatever you can conceive. Not on the telephone though.* (P5)

Another benefit of time and location flexibility is that there is no perceived need for disclosure of a patients’ participation in psychotherapy. This allows the patient to avoid social stigma associated with traditional face-to-face psychotherapy.

*“I am very busy in my job, I am in a leading position and it is rather difficult, if I - I would not be able to say that I will go to therapy in the afternoon. This would make them [co-workers] feel insecure, and I was really relieved, let`s say a win-and-win situation that I can do the therapy like this.“* (P8)

The omission of the periodic journey to the therapist allowed for a perceived anonymity, which facilitated the first step in starting psychotherapy.

#### Perceived difficulties with telephone (C3c2)

The flexible character of the treatment did not only result in perceived advantages. For some patients these exact aspects posed challenges in their experience of tel-CBT. However, most of the reported difficulties were practical in nature, such as finding an appropriate location for receiving the phone calls or making sure that the mobile phone is charged. Patients reported two challenges imposed by the telephone, which related to the therapeutic content: one patient observed that it was particularly challenging to convey emotions and feelings solely through words and descriptions alone, which resulted in only partially processing the mental state together with the therapist.

*“Maybe with the limitation – and that is probably part of the telephone-therapy – that the deepening of single emotional states and also expressing these, this was not very possible. It remained more on the cognitive level. And even though that was also helpful and accommodated me, but real feelings, which are associated with specific mental states, like anxiety or depressiveness, this maybe for me was not so easy to express.”* (P7)

The second difficulty emerged from the experience that the therapy taking place in the private environment involved a lack of distance from the problem area:

*“[ … ] in a regular counselling, well I don`t really know, but one also has fixed appointments. But one goes there and is basically out of the daily live, pulled out of what one is currently doing. The telephone conversations are more mood-dependent, because one does not prepare for it equally like going to the therapist, where one might get ready for it on the way there. Like this, the conversation comes bursting in and one then naturally has a snap-shot of the current mood.”* (P10)

#### Closeness versus distance (C3c3)

Properties ascribed to the telephone were placed between the poles of perceived (physical and emotional) closeness and distance.

*“I found it very special, because these telephone conversations do not take place in the therapist’s office, but in my own environment. And that`s what makes it more intimate, it makes the therapist be closer to me, strangely enough. ( … ) I am laying on my couch, within my own four walls and I perceive this as very close and personal. In fact, more personal than if I go to a therapist and sit decently on a chair. That for me is almost more distance.”* (P13)

While the implantation of the therapeutic interaction to the patients’ personal environment created a perceived emotional nearness to the therapist, the physical distance allowed for regulating the closeness. The interplay between closeness and distance is captured in the combination of being able to open up, yet deciding the extent of disclosure in the interaction with the therapist.

*“At some point I was somewhat sentimental and, in this moment, it was good for me to weep freely, without anyone seeing it ( … ). I think one could tell from my voice but you somehow do not need to show your face. This anonymity was pretty good in these moments. It is somehow not so embarrassing and one is protected by the telephone.”* (P4)

### Patients` conclusions and implications of low-intensity CBT for treatment path

#### Relevance of low-intensity CBT (C4c1)

Putting the experiences with the treatment in a wider context, one quintessence was the relevance of the low-intensity CBT for the personal treatment path. The impact of the therapy concept is ascribed to an immediate, and to a more comprehensive, overarching dimension. Parallel to the narratives on concrete CBT strategies, on the content level patients reported on a tangible, directly available, and individualised outcome resulting from the treatment:

*“In the end, I think it is about packing the tool box or so. Mine is probably poorly equipped, but I have the exact right tools, I believe.“* (P8)

More specifically, the relevance of low-intensity CBT as a whole is attributed to the insight of having achieved stability and a sense of control:

*“I have the feeling that I have unrigged the support wheels, I am actually riding freely.”* (P8)

On an overarching dimension, the narratives reveal a relevance on the long run, arising from the treatment’s capability of increasing the patients’ self-efficacy, also with an eye towards future difficult situations:

*“This is one main effect of this telephone-based therapy in fact … -support, and that it truly improved my own competency regarding coping with difficult mental states.”* (P7)

Some patients mentioned that the low-intensity CBT launched a reflexion processes and the transfer of insights to other areas of life.

*“Well I simply noticed that this is also something, that is not just for a short period of time and that you are done with it and can leave it aside ( … ) I think it requires a constant staying on the ball and practicing and making aware of.”* (P12)

Ultimately, for three patients with previously unsatisfying psychotherapy experience, the current treatment presented a corrective experience. For two patients, participating in tel-CBT served as a steppingstone to a regular, high-intensity evidence-based psychotherapy.

#### Ephemerality of low-intensity CBT (C4c2)

This category evolved due to expressed doubts regarding the sustainability of the treatment effect. Despite the perceived tangible and longer-lasting output of the guided self-help approach in form of the personalised workbook and acquired individual skills, some patients recognised that an enduring benefit depends on continuing work and usage of the tools. For these patients it is questionable whether the therapy length and dose is capable of solidifying the therapeutic effect.

*“What is difficult to say, is that you never know what happens in half a year or a year. Will it last? Or would one be happy to go back to it, to taking up the thread again. Or strengthening or so … I find that now actually a little bit of a pity that somehow … It somehow disappeared, or that it is vanishing.”* (P3)

The two patients who started high-intensity CBT after tel-CBT would have wished for a longer therapy in order to internalise strategies learned, particularly the cognitive part of the treatment.

#### Acceptance and satisfaction (C4c3)

Generally, there was a positive evaluation of the treatment concept, a high satisfaction with the performance of the therapists, with the CBT emphasis, and with the procedure in general.

*“I think there is a wide spectrum of people, who might benefit from such a treatment format, from this kind of support.”* (P3)

## Discussion

The overall purpose of the current process evaluation was to shed light on helpful factors inherent to guided self-help and factors associated with the telephone as a treatment medium, by qualitatively exploring patients’ views and experiences with a telephone-based guided self-help CBT. The content analytic techniques of structuring, summarising, and explicating revealed four category clusters, which subsume 15 categories.

The category cluster is constituted of expectations as well as fears toward the treatment both on a practical/executive and content level. Similar to findings from a qualitative study on expectancies in a minimal intervention [24], positive and negative expectations toward the treatment procedure and outcome were rather vague. Patients’ participation was largely driven by an appeal to the novelty of the therapy format and a general curiosity and interest in how therapy would work over the telephone. While a previous exploration of patients’ views on remote technology-based care [4] highlights the perceived accessibility enabled through the telephone, our data reveal that facilitated access to psychotherapy was just one of several reasons for starting this type of treatment. Patients’ narratives rather indicate the perception of being in the right place at the right time. It is important to note that in contrast to Bee et al.’s [5] study, our patient sample was exclusively self-referred, even though the RCT was set in routine care, meaning that all interviewed patients explicitly chose this treatment out of other alternatives available. Interestingly, three patients explicitly mentioned poor previous experiences with psychotherapy, which incited patients to try a new and seemingly less personal therapy.

The second category cluster contains therapist- and treatment-related aspects of guided self-help CBT, corresponding largely to specific and common factors in psychotherapy research. The exclusively positive qualities attributed to the therapists underline the significance of the therapists’ role in the guided self-help CBT. While we did not include standardised measures of the working alliance, parallels to Bordin’s [8] conceptualisation of the therapeutic alliance can be drawn. The initial feeling of safety and trust as well as the interpersonal connectedness reported by the patients correspond largely to the solid foundation of bonds as one facet of the therapeutic alliance. Parts of the emotional bond between therapist and patient was – in retrospective – afforded to the initial personal contact. Although this finding might support the assumption that therapeutic properties and contextual characteristics of the client-therapist interaction are contesting the legitimacy and quality of remote mental health consultation (May et al. [25]), it remains unclear whether the same level of trust and wellbeing would have emerged without the personal encounter at the beginning of the treatment. More importantly, the results show that patients varied in their perception of the personal encounter being an essential requirement for continuing the treatment with the same openness and comfort. Our data might support previous notions that the therapist contact per se is more important than whether the patient-therapist encounter is personal or not (Knaevelsrud & Maercker, 2007). However, since we were not able to compare patients’ experiences of tel-CBT with and without personal encounter, our results are unique to tel-CBT involving one initial face-to-face contact between therapists and patients.

A central treatment-related category encompasses patients’ narratives on CBT principles. In summary, the problem-focused approach and the emphasis on resolving current problems were deemed important determinants of a successful treatment. This contrasts previous findings on the evaluation of a low-intensity CBT, where patients would have preferred to understand the roots of their condition rather than battling current symptoms [24]. It might be that the rather intensive therapeutic contact in our study facilitated a sufficiently profound elaboration on patients’ history, which is also reflected in patients’ accounts on a flexible adjustment of the programme to the patients’ individual issues. Moreover, the fact that patients made sense of diverse therapeutic strategies (e.g., monitoring the interplay between mood, thoughts, and behaviours; having ready individual variants of cognitive restructuring techniques) points towards the therapists’ competency in delivering specific techniques. Additionally, the value placed on the therapists’ input within the therapeutic dialogue and the patients’ reliance upon the therapist bringing the contents of the workbook to life, testifies to the therapists’ expertise and the perceived importance of the therapists’ competency. It needs to be considered that the therapists in our study were clinical psychologists with advanced training in CBT in contrast to less specialised personnel (lay-therapists, study nurses, psychological well-being practitioner), which are commonly employed to deliver low-intensity CBT. The trade-off between the therapists’ level of expertise, the intensity and length of treatment, and the associated costs needs to be evaluated in additional studies.

Helpful “specific factors” in the treatment were primarily symptom monitoring and cognitive restructuring (e.g., recognising, challenging, and replacing irrational thoughts and beliefs). This result might be owed to the fact that our patient sample is middle-aged and predominantly shows mild to moderate depression severity, while behavioural activation has shown effectiveness in younger people [37] and more severely depressed patients [16]. The patients in our study show a comparatively high level of functioning, which is reflected in the high employment rate (70%) of the interviewed sample and the generally high level of social and physical engagement in both the employed and retired patients. It is possible that patients did therefore not perceive additional benefit in scheduling pleasant activities. Monitoring symptoms and reflecting on thought patterns, on the other hand, might have provided more tangible and new skills to understand and manage depressive symptoms. Future research might focus on the role and interrelation of symptom monitoring, cognitive work, and behavioural activation in low-intensity interventions. Ultimately, encouraging patients to become “experts” on themselves and to find ways to help themselves was one product of tel-CBT. These findings largely correspond to qualitative studies on experiences with traditional CBT suggesting that specific techniques as well as common psychotherapeutic ingredients are important from the patients´ point of view [13, 36]. The engagement with the workbook was placed at the core of the treatment. All patients reported of having used the workbook regularly during treatment, and most of them felt comfortable with writing down their feelings and thoughts. It needs to be noted that our patient sample is highly educated, so while writing down thoughts and reflections is feasible and helpful, generalisability is not possible; other patient populations (e.g. more severely depressed ones) might have more difficulties with written material so that personal therapeutic guidance might be even more important. Despite emphasising the workbook as a concrete and helpful aspect of the treatment, the accounts in this category illustrate that much of the workbook’s impact was enabled by the therapist. This finding corresponds largely to a growing body of evidence on the impact of therapeutic homework on treatment effect (e.g., [21]). Within the homework literature single studies have demonstrated that specific homework-related therapist behaviours lead to increased homework engagement on the patient side [11, 14]. However, there is a lack of studies, which directly compare the influence of therapist support and autonomous engagement with self-help activities on patients’ homework compliance or on treatment outcome. Our finding that CBT-techniques found complete expression in the synergy of workbook and therapist guidance might inform future studies, which intend to empirically test the relationship between therapist behaviours regarding homework assignment and review, patients’ homework engagement, and treatment outcome.

The third category cluster arose from reported facets relating to the telephone as a treatment modality. The discourse on the treatment medium was characterised by a perception of establishing closeness in the absence of physical proximity [5], which highlights the capacity of the telephone to convey central therapeutic “ingredients” (in this case psychological closeness). Moreover, the value assigned to therapist skills and qualities, as indicated by previously reported categories, shows that it is possible to mediate aspects of a working alliance remotely, identical to previous conclusions (e.g., [5]).

Challenges that are often linked to the medium telephone – such as restricted communication due to missing visual cues – did not come true. In addition, negative effects connected to the therapy taking place in the home setting (e.g., distraction) were not confirmed. Although one patient pointed out the potentially intruding character of the telephone calls, this was not mentioned as being problematic by others. Interestingly, most of the interviewed patients reported the opposite; that the mediation of therapy content by the telephone helped focusing on therapy contents. The content dimension was placed at the centre of the therapeutic interaction while the interpersonal dimension stayed in the background. This also implies that telephone-based treatments might not be the most suitable options for patients with interactional difficulties.

The fourth category cluster comprises implications for the patients’ treatment pathways. One conclusion touched upon the ephemerality of the treatment effect. Some patients expressed doubts about the sustainability and long-term effects of the low-intensity CBT. While some patients pointed out the necessity to practice and internalise acquired skills in more depth, there is also quantitative evidence on the inconclusive long-term effect on symptomatology, with one study demonstrating high relapse rates in people completing low-intensity CBT [1]. However, more investigations of patients` pathways with a long-term perspective are necessary. The category satisfaction and acceptance emerged due to a generally positive evaluation of all treatment components and the treatment concept as a whole. While this finding goes in line with previous positive evaluations and corresponds to low dropout rates in tel-CBT [28], there might be other reasons involved, such as low expectancies towards the treatment. It could be that on the one hand patients were positively surprised by the therapeutic comprehensiveness of what might have first appeared as a simple telephone-counselling, while on the other hand previous negative experiences with psychotherapy (reported by some study participants) might have set low expectations toward the treatment in the first place.

Several limitations warrant further discussion: First, we would like to point to problems at the core of the qualitative methodological approach, such as the question of whether we could reach saturation of data. Although the sample was broadly representative of the study participants of the RCT in terms of sociodemographic and clinical characteristics, we were not able to include patients who were not or only partially motivated for treatment due to their self-referral to the study. It would be interesting and important to explore patients’ experiences and views when referred by GPs or other providers. Given the suitability of low-intensity CBT to be integrated into primary care, experiences of patients who did not actively seek this type of treatment, would allow for more externally valid results. However, the reports tended to be homogeneous within the interviewed sample and repetitive within the individual interviews after completing the interview guide, which is indicative of data saturation. Second, the predominantly positive evaluation of the intervention might have been influenced by a socially desirable response style. We tried to circumvent response bias by assigning an independent member of the study team as interviewer. Speaking about experiences in an interview might, however, still be more inhibiting compared to questionnaire-based items on satisfaction with the treatment. Third, although the study`s purpose was to differentiate between helpful aspects specific to the guided self-help approach from distinct properties of the treatment delivery medium, this type of data and analysis does not allow to draw definite conclusions about the relative impact of each component.

## Conclusion

In summary, interviewed patients report positive experiences with this type of low-intensity CBT touching on structure, content, and procedure. While critical evaluations are related to practical aspects of tel-CBT, helpful facets are mostly afforded to the dimension of the treatment modality, the support by the therapist, and the interplay of those. We believe that the combination of professional guidance and self-help represents an appropriate balance between therapeutic input and patient’s self-reliance for individuals in need of care.

## Additional file

Additional file 1: Interview guide. (DOCX 22 kb)

## Data Availability

The datasets generated and/or analysed during the current study are not publicly available due to confidentiality, but are available from the corresponding author on reasonable request and pending ethics clearance from KEK Zurich following this request.

## Abbreviations

CBT: Cognitive behavioural therapy
GP: General Practitioner
IAPT: Improving Access to Psychological Therapy
ICD: International Classification of Diseases
PHQ-9: Patient Health Questionnaire 9
RCT: Randomised controlled trial
tel-CBT: Telephone-based cognitive behavioural therapy
