# Supplementary material for: “Unrigging the support wheels” - A qualitative study on patients’ experiences with and perspectives on low-intensity CBT
Source: BMC Health Serv Res. 2019 Oct 9;19:686. doi: 10.1186/s12913-019-4495-1 (PMC6784338; doi:10.1186/s12913-019-4495-1)
Supplement: Supplementary file 1 — Interview guide. (DOCX 22 kb) [file 12913_2019_4495_MOESM1_ESM.docx]

Additional File 1

Interview guide

| **Reasons for starting treatment**  To start off, I would like to know how you accessed the telephone-delivered treatment. | |
| --- | --- |
| 1. How did you learn of the telephone-delivered psychotherapy (tel-PT)? | |
| 1. Which reasons existed for starting a telephone-delivered psychotherapy? | |
| Prompt/Follow-up | - Why did you choose tel-PT over a different treatment? - Did you plan to follow-up a previous treatment? |
| 1. How would you generally describe your experience with tel-PT? *(see question 15)* | |
| Prompt/Follow-up | - *Continuing with question 6, 7, 13* |
| **Medium telephone**  The next part is about the telephone as a means of communication and how you perceived the treatment delivered over the telephone. | |
| 1. Which expectations and conceptions about a treatment mediated by the telephone did you have prior to starting? | |
| Prompt/Follow-up | - Were there further expectations? - Did you have further fears? - Were the expectations met or disappointed? |
| 1. How did you generally feel about talking on the phone? | |
| Prompt/Follow-up | - Were there any special features about talking on the phone? - Were there any advantages / disadvantages? |
| **Telephone-Psychotherapy – Structure**    We would also like to know how you got along with the structure of the treatment programme. This includes aspects such as time and duration of the treatment as well as content and procedures within and between the treatment sessions. | |
| 1. The treatment consisted of one personal clinical assessment and a limited amount of weekly and biweekly telephone sessions. How did you perceive the format and the extent of the entire treatment? | |
| 1. Within the sessions some topics were presented by the therapist, whereas other topics were meant to be worked on independently, in form of readings and exercises. How did you perceive this procedure? | |
| Prompt/Follow-up | - What do you think about the combination? - Which parts did you perceive helpful? - Which parts did you perceive difficult? |
| **Telephone Psychotherapy – Content** | |
| In the next section I will ask you about the content of the treatment and I am curious about what you thought of the contents and whether there was something helpful or not so helpful about it. | |
| 1. You were informed prior to the study that the treatment will be a cognitive behavioural therapy. Which expectations did you have regarding the content of this treatment? | |
| Prompt/Follow-up | - Did you know what CBT was prior to starting? Any experience? |
| 1. Which content or which aspects of the treatment did you perceive helpful? | |
| Prompt/Follow-up | - Why? Can you give a reason / example for that? - Were there any strategies that you remember? Examples? |
| 1. How did you feel about reading in the workbook and working with the worksheets between the sessions? *(if not answered in question 7)* | |
| Prompt/Follow-up | - What do you think about the treatment alignment on the workbook? |
| 1. Were you able to realise the therapeutic content in your daily life outside of therapy sessions? | |
| Prompt/Follow-up | - Independent activity – what is your attitude / conclusion / result? - Were there any factors that helped / hindered independent activity? |
| 1. After having finished the treatment – how do you feel about the content now? | |
| Prompt/Follow-up | - Which role – if any – does tel-PT play today? - Are activities and strategies used in daily life? |
| **Therapist** | |
| It is interesting for us to hear how you perceived working with your therapist and what you thought of the therapist. | |
| 1. How did you perceive working with the therapist? | |
| Prompt/Follow-up | - How did you feel about working with the therapist? - What role played therapist in the treatment for you? |
| 1. How was it for you to meet the therapist in one personal session? | |
| Prompt/Follow-up | - How would you describe the association between the personal meeting and the subsequent sessions? |
| **Final questions** | |
| We now come to the last questions. I would like to know more about your overall evaluation of tel-PT. You will also be able to provide final comments. | |
| 1. How would you generally evaluate tel-PT as a whole? *(see question 3)* | |
| Prompt/Follow-up | - What is your conclusion? - Would you recommend tel-PT to a good friend? - Was there any situation or moment when you thought about stopping the treatment? For which reason? - Is there anything you would have wished differently? |
| 1. When you are thinking of your treatment path – which role did tel-PT play? | |
| Prompt/Follow-up | - Where do you currently stand? - Do you feel like you would need further therapeutic treatments? - Would you engage in a telephone-based treatment again? |
| **Is there anything else you would like to say?**  **Thank you very much for your time and your openness during this interview.** | |
